# Evaluation of ascorbic acid as an intervention of metal toxicity in dogs in Kabwe district

**DOI:** 10.1016/j.vas.2025.100519

**Published:** 2025-10-09

**Authors:** Nelly Banda, Mahongo Selwa, Rio Doya, Nyein Chan Soe, Andrew Kataba, John Yabe, Golden Zyambo, Kaampwe Muzandu, Yared Beyene Yohannes, Yoshinori Ikenaka, Mayumi Ishizuka, Shouta MM Nakayama

**Affiliations:** a Laboratory of Toxicology, Department of Environmental Veterinary Sciences, Faculty of Veterinary Medicine, Hokkaido University, Kita 18, Nishi 9, Kita-ku, Sapporo 060-0818, Japan; b School of Veterinary Medicine, The University of Zambia, Lusaka P.O. Box 32379, Zambia; c Department of Pharmacology and Parasitology, University of Veterinary Science, Yezin, Nay Pyi Taw, 150501, Myanmar; d School of Veterinary Medicine, University of Namibia, Windhoek P/B. 13301, Namibia; e Water Research Group, School of Environmental Sciences and Development, North-West University, Potchefstroom 2531, South Africa; f Translational Research Unit, Veterinary Teaching Hospital, Faculty of Veterinary Medicine, Hokkaido University, Sapporo 060-0818, Japan; g One Health Research Centre, Hokkaido University, Sapporo 060-0818, Japan

**Keywords:** L-ascorbic acid, Pb, Dogs, Oxidative stress, Male, Female

## Abstract

Non-essential metals and metalloids are known to induce oxidative stress in exposed organisms, often leading to cellular damage and systemic toxicity. While chelation therapy remains the primary treatment for metal toxicity, its application is limited by side effects. L-ascorbic acid (L-AA), a widely available antioxidant, has emerged as a promising nutritional intervention for mitigating metal-induced oxidative stress. Dogs, whose blood lead levels (BLLs) closely mirror those of humans, have been utilized as sentinel species in environmental toxicology studies.

This study aimed to evaluate the therapeutic potential of L-AA in dogs residing in Kabwe, Zambia, a former mining town where remediation of heavy metal contamination is ongoing. The reported BLLs in Kabwe dogs ranged from 0.43 µg/dL to 123.5 µg/dL. A total of 22 dogs (10 females and 12 males) received oral L-AA supplementation daily for 14 days. Blood samples were collected on Day 1 of L-AA administration and Day 14 to assess biochemical and toxicological changes.

Post-treatment analysis revealed statistically significant reductions in plasma malondialdehyde, cortisol, blood urea nitrogen, and creatinine levels, as determined by Student’s t-test and Wilcoxon signed-rank test. Additionally, δ-aminolaevulinic acid dehydratase activity was significantly elevated, indicating improved oxidative status. These findings support the efficacy of L-AA in attenuating oxidative stress associated with metal and metalloid exposure, even in the absence of exposure cessation.

Interestingly, Pb levels declined predominantly in dogs younger than 24 months. Furthermore, reductions in Zn and Cu commonly linked to L-AA administration were observed exclusively in male dogs, suggesting a sex-specific response.

## Highlights

• Oxidative stress biomarkers were reduced in dogs after administration of L-ascorbic acid.
• The reduction of BLL occurred mostly in younger dogs under 24 months of age.
• The reduction of Pb, AS, and Cd occurs in both males and females.
• The reduction of essential metals, Cu and Zn, occurs in male dogs but not in females post-treatment with L-ascorbic acid.

## Graphical abstract

**Figure:**
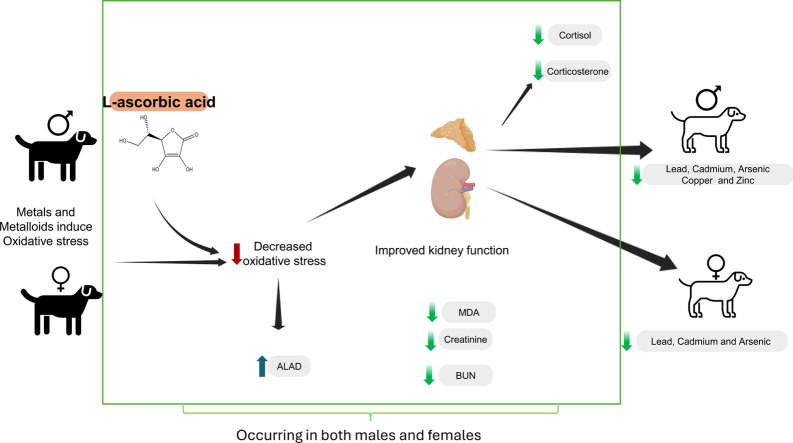

## 1 Introduction

Lead (Pb) is among the most prevalent metal contaminants in the world. It is a problem due to its inability to biodegrade in the environment and affects many organ systems in the body; including neurological, haemopoietic, nephrological, and reproductive systems (Tangahu et al., 2011; Wani et al. 2015).

Oxidative stress is one of the main mechanisms by which Pb causes toxic effects in the body (Dobrakowski et al., 2017;Lee et al., 2019). However, redox reactions maintain normal homeostasis; without this, reactive oxygen species will react with various macromolecules in the body and damage them. Deoxyribonucleic acid (DNA), when oxidised, will result in the decay and instability of the genome. Oxidative stress has been linked with cancer development, cognitive impairment, progression of chronic kidney disease, chronic obstructive pulmonary disease, diabetes, cardiovascular disease, aging, and aging-related diseases (Liguori et al., 2018; Sies, 2020; and Sies et al., 2025).

Treatment options for Pb toxicity are limited, with chelation therapy as the mainstream choice of treatment (Smith & Strupp, 2013). Ethylenediaminetetraacetic acid (EDTA), dimercaptosuccinic acid (DMSA), and other drugs are used to bind to Pb in the bloodstream after intravenous or oral administration, and this allows Pb to be excreted via urine (Aaseth et al., 2018; Cao et al., 2015; Van Eijkeren et al., 2017; Li et al., 2023). There are a few downsides to chelation therapy; for best results, sources of exposure must be identified, and exposure should be stopped. This is not always possible for individuals living in Pb-contaminated areas. Additionally, chelation therapy has been known to cause side effects to the body which may include neuropathy, diarrhoea, vomiting, nausea, body rashes, fevers, pruritis, and increased heart rate (Kim, Kim and Kumar, 2019; Li et al., 2023).

Dogs have been proven to be used as sentinels for Pb exposure in polluted areas (Beck et al., 2020; Chen et al., 2023; Hegedus et al., 2023; Pastorinho & Sousa, 2020). A study conducted in the Ionian Etnean volcanic region used dogs to investigate whether living in that area affected the accumulation of metals in their bodies. This is because dogs share environments with humans and have similar organ structures (Bruno et al., 2025). Although it has also been documented that metal exposure in dogs affects various organs, poisoning in dogs from metals such as Pb usually goes unrecognized by veterinarians (Assi et al., 2016;Gori et al., 2021;Langlois et al., 2017). Moreover, due to the overall cost of chelation therapy, dogs may not be able to receive the same treatments as humans.

L-ascorbic acid (L-AA), also known as Vitamin C, functions as an antioxidant by slowing or preventing the oxidation of various substrates. The use of antioxidants to combat oxidative stress has been shown to ameliorate the effects of Pb in rodent models and human studies, showing improvements in fertility and neurological functions (Abam et al., 2008; Ahmad et al., 2020; El-Sebeay, Ibrahim and Yousif, 2017; Ghafouri-Fard et al., 2021; Kim and Kang, 2017; Nam et al., 2018; Nam et al. 2019; Raeeszadeh et al., 2021;Rendón-Ramírez et al., 2014). Although not directly used for alleviating Pb toxic effects, it has been used in dogs during disease and other non-physiological states to support body functions (Bandodkar et al., 2016; Polymeropoulos et al., 2016; Rizk et al., 2017; Sechi et al., 2017; W. Wang et al., 2016).

Antioxidants have been utilized in several studies to treat metal toxicities. However, their effectiveness in dogs with elevated Pb levels and other metal toxicities remains unclear. The primary objective of this study is to evaluate the use of L-AA in reducing oxidative stress in dogs living in metal-contaminated areas, without interrupting their ongoing exposure. To assess the efficacy of L-AA as a treatment option, changes in metal and metalloid concentrations, δ-aminolaevulinic acid dehydratase (δ-ALAD) activity, cortisol, blood urea nitrogen (BUN), creatinine, and malondialdehyde (MDA) levels before and after treatment were analysed. Successful treatment was expected to result in reductions in oxidative stress-related biomarkers, including δ-ALAD, MDA, and cortisol.

## 2 Materials and methods

This study received clearance from the Zambian Ministry of Fisheries and Livestock and was carried out strictly per its regulations. Ethical approval clearance was obtained from the University of Zambia Biomedical Research Ethics Committee (UNZABREC) under reference number 5310-2024.

### 2.1 Sampling area

Kabwe (14.4285° S, 28.4514° E) is a former mining town in Zambia known for its severe environmental contamination from heavy metals. For this study, the townships of Kasanda and Chowa were selected based on previously reported concentrations of metals and metalloids in dogs, as documented by Soe et al., (2024) and Toyomaki et al., (2020). According to Toyomaki et al., 2020, the average BLL in dogs from Kasanda was 52.5 µg/dL, while those from Chowa averaged 24.7 µg/dL.

Environmental contamination in Kabwe soils is marked by high levels of several toxic metals and metalloids. Reported concentrations include arsenic (As) ranging from 0.57 to 35 mg/kg, cadmium (Cd) from 0.030 to 17 mg/kg, copper (Cu) from 7.3 to 296.7 mg/kg, lead (Pb) from 6.7 to 23,798 mg/kg, and zinc (Zn) from 60 to 13,133 mg/kg. These values often surpass internationally accepted soil quality standards, which specify limits of 1–15 mg/kg for As, 0.07–11 mg/kg for Cd, 6–60 mg/kg for Cu, 10–70 mg/kg for Pb, and 17–125 mg/kg for Zn (Doya et al., 2020).

As part of ongoing remediation efforts in Kabwe’s high-risk residential areas, the Zambia Mining and Environmental Remediation and Improvement Project, supported by the World Bank Group, has implemented soil paving around households to reduce exposure to contaminated dust (World Bank Group, 2020). For this study, subjects were selected from households with both paved and unpaved yards to assess the potential impact of this intervention on environmental exposure.

#### 2.1.1 Inclusion criteria

• All healthy dogs living in the above townships from either a household with a paved yard or not.

#### 2.1.2 Exclusion criteria

• Dogs that present with clinical signs of disease upon completion of clinical exams (fever, pallor, heart murmurs, excessive ectoparasites, poor body condition)
• Dogs that were apprehensive about being handled by investigators, due to the potential of handling causing stress.

After obtaining owner consent, 30 dogs residing in the designated townships were enrolled in the study. This sample size was selected as it was both sufficiently large and ethically appropriate. During the initial health check-up, 8 dogs were excluded: 6 exhibited aggressive behaviour and could not be examined by the investigators, while 2 required medical attention due to poor body condition (emaciation).

The remaining 22 dogs received L-AA at a dosage of 50 mg/kg/day for 14 consecutive days. The total daily dose of L-AA administered ranged from 250 mg to 1000 mg per dog for each day. Dosage levels administered in this study were determined based on recommendations and findings from a comprehensive review by Gordon et al., (2020). Of these 22 dogs, 10 were female and 12 were male, with mongrels being the predominant breed. Other breeds included Boerboel, Maltese, and German Shepherds (Table 1). All dogs came from households with similar income levels and were fed comparable diets.

**Table 1:** Presents the characteristics of study participants, including breed, age, sex, and body condition score.

Subject	Breed	Age (Months)	Sex	Body Condition Score (5)
Subject 1	Mongrel	48	Female	2
Subject 2	Boerboel	8	Male	4
Subject 3	Boerboel Cross	8	Male	3
Subject 4	Boerboel cross	48	Female	3
Subject 5	Mongrel	5	Female	1.5
Subject 6	Mongrel	16	Male	2
Subject 7	Maltese Cross	18	Female	2
Subject 8	Boerboel	8	Female	3.5
Subject 9	Mongrel	24	Male	2
Subject 10	Toy Maltese	60	Female	3
Subject 11	Mongrel	38	Female	3
Subject 12	Mongrel	8	Male	3
Subject 13	Mongrel	24	Male	3
Subject 14	Boerboel Cross	48	Male	3
Subject 15	German Shepherd Cross	24	Male	3
Subject 16	Mongrel	24	Male	3
Subject 17	Mongrel	36	Male	1.5
Subject 18	Mongrel	36	Female	3
Subject 19	Mongrel	36	Male	3
Subject 20	German Shepherd	72	Female	3
Subject 21	Mongrel	36	Male	3
Subject 22	Mongrel	7	Female	3

The duration of administration was based on the protocol established by Abam et al., (2008). Blood samples were collected on the first day of L-AA administration and again on Day 14. To minimize stress from excessive handling, L-AA tablets were given early each morning, concealed within a treat (typically a piece of sausage). Daily health checks were conducted to monitor any changes in the dogs throughout the study period. These assessments included recording physical parameters and asking owners about any noticeable changes in stool appearance.

After the collection, the blood was separated into whole blood, serum, and plasma, and thereafter, was frozen and sent to the University of Zambia, School of Veterinary Medicine, Department of Biomedical Sciences. Then, it was stored at -80°C and transported to the Laboratory of Toxicology, Faculty of Veterinary Medicine, Hokkaido University for further analysis.

### 2.2 Metal and metalloid concentration

For the determination of metal concentration, 100 µl of blood and 5ml of 30% nitric acid (Cica reagent, Specific gravity of 1.38, 60%; Kanto Chemical Corp., Tokyo, Japan) and 1 ml of hydrogen peroxide (Cica reagent, 30%; Kanto Chemical Corp.) were added into a microwave vessel. These were then digested in a speed wave microwave (Berghof, Eningen, Germany). Inductively coupled mass spectrometry (ICP-MS, 7850 series; Agilent Technologies, Tokyo, Japan) was used to measure the metal and metalloid concentrations: As, Cd, Zn, and Pb.

Analytical quality control was conducted using Seronorm^TM^ (Trace Elements Serum; SERO, Norway), and the recovery rates in replicate measurements yielded were 86%-94% for metals As, Cd, Cu, Pb, and Zn.

### 2.3 Malondialdehyde assay

Fifty microlitres of blood plasma samples were collected to quantify malondialdehyde (MDA) levels as a biomarker of oxidative stress, both prior to treatment and on Day 14 post-treatment. MDA concentrations were measured using the Assay Genie® competitive MDA ELISA (enzyme-linked immunosorbent assay), following the manufacturer’s protocol. All samples were analysed in duplicate to ensure the reliability and reproducibility of the results.

### 2.4 δ-aminolaevulinic acid dehydratase activity

The δ-aminolaevulinic acid dehydratase activity assay was conducted following the methodology described by Maruyama et al., (2024), with minor modifications. Two aliquots of 20 μL whole blood from each subject were prepared to measure non-activated and reactivated δ-ALAD activity.

For the non-activated assay, 80 μL of 0.1% Triton X-100 (Sigma–Aldrich, MO, USA) was added to lyse the blood sample. Subsequently, 100 μL of 0.5 M morpholinoethanesulfonic acid (MES) buffer (Tokyo Chemical Industries, Tokyo, Japan), 50 μL of 60 mM 5-aminolevulinic acid (ALA) hydrochloride (Sigma–Aldrich) in PBS, and 50 μL of distilled water were added. For the reactivated assay, the same volumes of Triton X-100, MES buffer, and ALA hydrochloride were used, followed by the addition of 25 μL of 0.8 mM zinc acetate and 25 μL of 1 M dithiothreitol (DTT) (both from Himedia Laboratories Pvt. Ltd., Mumbai, India). While the blank samples were prepared with 80µl of Triton X-100, 100µl MES buffer, and 50 μL of distilled water.

After a 60-minute incubation at 37 °C, the reaction was terminated by adding 200 μL of a stop solution containing 0.4 M trichloroacetic acid (TCA; Merck, Darmstadt, Germany) and 60 mM mercury chloride (HgCl₂; Himedia Laboratories Pvt. Ltd.). This step was applied to both non-activated and reactivated assays. Samples were then centrifuged at 15,000 × g for 5 minutes, and 400 μL of the supernatant was transferred to new tubes. Each was mixed with 750 μL of modified Ehrlich’s reagent, composed of dimethylaminobenzaldehyde (Nacalai Tesque, Kyoto, Japan) in glacial acetic acid (99.6%; Himedia Laboratories Pvt. Ltd.) and perchloric acid (Merck KGaA, Darmstadt, Germany).

After a 10-minute reaction period, absorbance was measured at 555 nm using a UV spectrophotometer (Shimadzu UV-2600, Shimadzu Inc., Kyoto, Japan), with appropriate blanks. Enzyme activity was expressed as μmol porphobilinogen (PBG) produced per hour per liter of red blood cells.The δ-ALAD activity was then expressed as μmol PBG/h/L blood using the following equation.

μmolPBG/h/Lblood=dilutionfactor×(PBGabsorptivity)

The difference between the native δ-ALAD activity and the activity of the blank sample was then used as δ-ALAD activity in this study.

### 2.5 Cortisol measurement

#### 2.5.1 Sample

A 50 uL aliquot of plasma was pipetted into a microcentrifuge tube, followed by the addition of 50 µl of 10 ppb internal standard (d4-cortisol). Next, 150 µl of 1 % of formic acid in acetonitrile and 250 µl of 80 % of acetonitrile (ACN) in distilled water (DDW) were added. The tube was then vortexed for 3 min and centrifuged at 10,000 x g for 10 min. For solid phase extraction, the supernatant was transferred to a Captiva EMR-lipid cartridge and passed through. To this cartridge, 300 µl of 80 % of ACN in DDW was passed through and the resulting solution was received into new glass tube. Then the solution was dried under vacuum and reconstituted using 150 µl of 0.1% formic acid in 60% methanol. The mixture was vortexed well and passed through a centricut filter before being transferred to LC vial.

#### 2.5.2 Instrumental analysis

An Agilent 1200 series UPLC system equipped with a Kinetex biphenyl column 150  ×  3.0 mm, 2.6 µm particle size (Phenomenex) was used for chromatographic separation. The mobile phase consisted of 0.1% formic acid in water (Phase A) and 0.1% formic acid in methanol (Phase B). The following gradient elution was used: 45% B from 0 to 0.50 min, increasing linearly to 88% B at 21.0 min, 100% B at 21.5 min, and kept at this concentration for 2 min before returning to the initial condition of 45% B at 23.01 min with a flow rate of 0.6 ml/min. The column was then conditioned with 45% B for about 3 min, giving a total run time of 26 min. The column was kept at a constant temperature of 50 °C during analysis and the injection volume was 30 µL. The injection syringe was washed between injections with 100 μl of solution containing equal parts of water and methanol and equal parts of isopropanol and acetonitrile. The injection interval was 1.5 min.

Quantification of steroids was done in the positive ion mode using an Agilent 6495 triple quadrupole LC/MS/MS system equipped with Agilent Jet Stream Electrospray ionization technology. The optimized parameters for the detection method are as follows: gas temperature of 290 °C, gas flow of 12 L/min, nebulizer gas pressure of 35 psi (2.4 bar), the capillary voltage of positive 3000 V; negative 2,500 V, and nozzle voltage of positive 0 V; negative 500 V. Analyses were conducted in the positive ionization mode and nitrogen was used as both nebulizing and drying gas. Quantitative analysis was performed in the multiple reaction monitoring (MRM) mode. The MRM transitions monitored were *m*/*z*: 363.2 to 327 (collision energy (CE = 12V) and 363.2 to 121.1 (CE = 24V) for cortisol, and 367.4 to 331 (CE = 13V), and 367.4 to 121 (CE = 26V) for d4-cortisol. The former product ions were used as quantitative, while the latter product ions were used as qualitative. Agilent Mass Hunter workstation software was used for all data acquisition and analysis.

### 2.6 Blood urea nitrogen (BUN) and creatinine (CRE) measurement

Plasma concentrations of blood urea nitrogen (BUN) and creatinine (CRE) were determined using the DRI-CHEM NX700 analyzer (Fujifilm, Japan), following the manufacturer’s protocol. For each measurement, 300 μL of plasma was used, ensuring that samples were free of bubbles and precipitation prior to analysis. Fujifilm DRI-CHEM reagent slides specific for BUN and CRE were inserted into the analyzer, and the measurements were automatically initiated.

### 2.7 Statistical analysis

Statistical analyses were conducted using JMP version 17. Depending on the data distribution, either the Student’s t-test (for parametric data) or the Wilcoxon test (for non-parametric data) was applied. Results are presented as mean ± standard deviation, and differences were considered statistically significant at p-values less than 0.05.

## 3 Results

### 3.1 Metal and metalloid concentrations

**Fig. 1:**
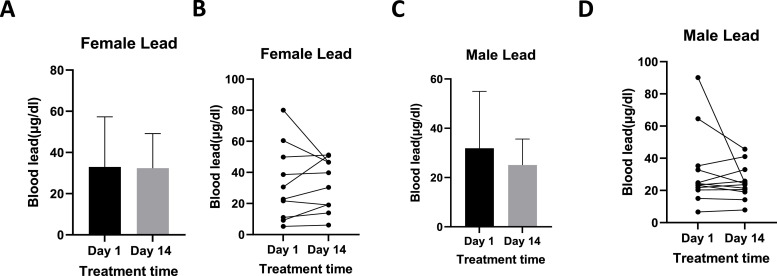
Shows Pb levels pre- and post-treatment in females (Fig. 1A and B) and in males (Fig. 1C and D). Fig. 1A and C show mean Pb blood levels, while Fig. 1B and D show individual responses. Females n =10, Males *n* = 12.

In females, Blood Lead Levels (BLL) ranged from 5.28 µg/dl to 80.01µg/dl pre-treatment to 6.09µg/dl to 51.19 µg/dl post-treatment (Fig. 1A and B). In males, BLL ranged from 6.61 µg/dl to 90.15 µg/dl on day 1 of treatment and from 7.88 µg/dl to 45.59 µg/dl on day 14 (Fig. 1C and D).

When Pb levels were compared between males and females, there was no statistical difference in Pb levels on day 1 and day 14; the P-values were 0.228 and 0.923, respectively. Henceforth, we combined the Pb levels from males and females and analysed the following after confirming that sex did not affect Pb levels in this study: Pb levels according to whether the yard is paved or not, Pb levels by age, and change in BLL Vs initial BLL.

**Fig. 2:**
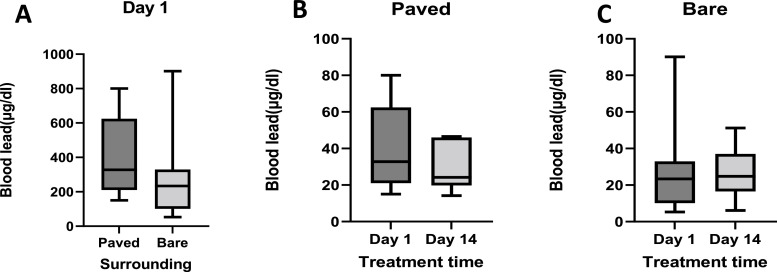
Shows Pb concentrations on day 1(Fig. 2A) before treatment in paved (n=9) and unpaved yards (*n* = 13). Fig. 2B shows dogs from homes with paved yards, and Fig. 2C shows dogs from homes where the yard is bare and how Pb concentrations change post-treatment.

Blood lead levels on day 1 ranged from 15.08 µg/dl to 80.01 µg/dl, while measurements taken on day 14 ranged from 14.18 µg/dl to 46.54 µg/dl. There was no statistically significant difference between the accumulation of Pb on day 1 of treatment in dogs living in paved and bare yards (Fig. 2A).

Although the median and standard deviation of the BLL were reduced, there was no statistically significant difference between samples collected on day 1 before treatment and those on day 14 from dogs living in paved yards (Fig. 2B).

**Fig. 3:**
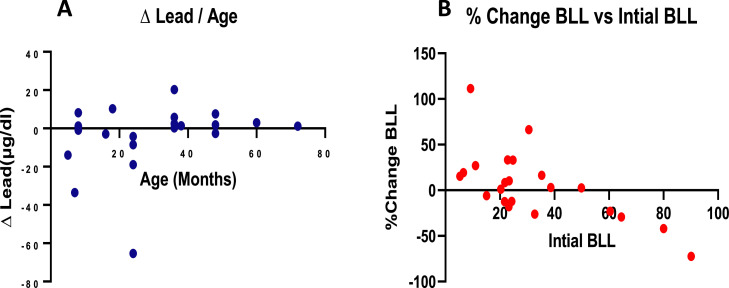
Shows Pb concentrations in dogs used in this experiment, Fig. 3A shows the change in lead concentrations by the subject's age, where ∆ lead = lead Concentration (Day 14 - Day 1). Fig. 3C shows the percentage change in BLL by the initial BLL, where % Change = (Day14-Day1)x 100. *n* = 22.

For dogs living in yards that were bare and unpaved, the range for BLL was 5.28 µg/dl to 90.15 µg/dl on day 1, and those on day 14 were from 6.09 µd/dl to 51.19 µg/dl. There was a reduction in the standard deviation and the upper extreme of BLL on day 14 (Fig. 2C). Overall, BLL levels reduced post-treatment (Fig. 1B and D); however, most reductions were recorded in dogs below 24 months old (Fig. 3A). The most reductions occurred in individuals who also had the highest BLL before treatment (Fig. 3B).

**Fig. 4:**
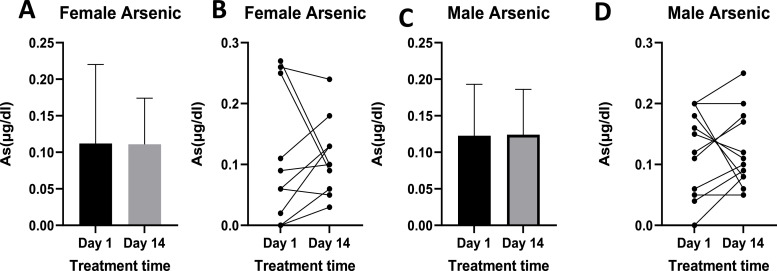
Illustrates As levels pre- and post-treatment in females (Fig. 4A and B) and males (Fig. 4C and D). Fig. 4A and C display mean As blood levels, while Fig. 4B and D present individual responses. Females *n =* 10, Males *n =* 12.

Arsenic levels in females and males pre-treatment ranged from 0.01 to 0.27 µg/dl and 0.01 to 0.20 µg/dl, respectively (Fig. 4). Post-treatment arsenic levels in females ranged from 0.01 to 0.24 µg/dl, while those in males ranged from 0.05 µg/dl to 0.25 µg/dl. Blood arsenic levels were reduced in some individuals (Fig. 4B and D), and the median, lower extreme, and upper extreme reduced post-treatment (Fig. 4B) in females. In both males and females, differences between day 1 and day 14 were not statistically significant.

**Fig. 5:**
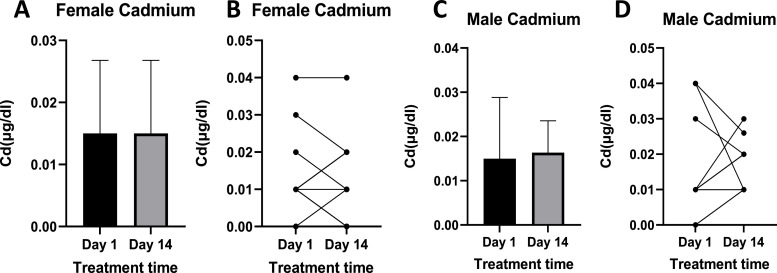
Shows Cd levels pre- and post-treatment in females (Fig. 5A and B) and in males (Fig. 5C and D). Fig. 5A and C show mean Cd blood levels, while Fig. 5B and D show individual responses. Females n =10, Males *n =* 12.

Cadmium levels were the lowest compared to other metals measured in this study (Fig. 5), with levels in both males and females being below 0.04 µg/dl. Although some reductions were recorded post-treatment (Fig. 5B and D), mean levels of Cd remained the same in females (Fig. 5A) or were slightly elevated post-treatment in males (Fig. 5C).

**Fig. 6:**
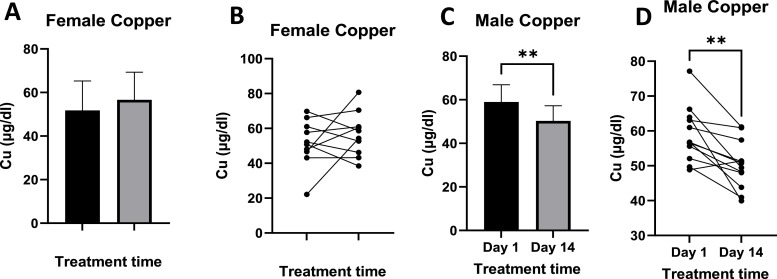
Shows Cu levels pre- and post-treatment in females (Fig. 6A and B) and males (Fig. 6C and D). Fig. 6A and C show mean Cu blood levels, while Fig. 6B and D show individual responses. Females n =10, Males *n =* 12. Where p-value ** = 0.01.

Copper levels in females ranged from 22.11 µg/dl to 61.09 µg/dl pre-treatment, and from 38.46 µg/dl to 80.70 µg/dl post-treatment (Fig. 6A). Levels of Cu in females increased after treatment with L-AA. In Males, however, levels of Cu are statistically significantly reduced, with levels ranging from 49.68 µg/dl to 77.13 µg/dl pre-treatment to 39.92 µg/dl to 61.13 µg/dl post-treatment.

**Fig. 7:**
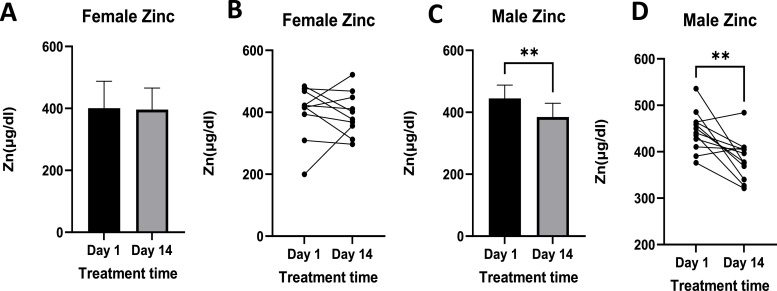
Shows Zn levels pre- and post-treatment in females (Fig. 7A and B) and males (Fig. 7C and D). Fig. 7A and C show mean ± standard deviation Zn blood levels, while Fig. 7B and D show individual responses. Females n =10, Males *n =* 12. Where p-value ** = <0.01.Data is expressed as mean ± standard deviation.

Zinc levels followed the same trend, with statistically significant reductions occurring in males but not in females (Fig. 7). In males, pretreatment levels ranged from 376.08 µg/dl to 535.76 µg/dl, while posttreatment levels ranged from 321.07 µg/dl to 484.14 µg/dl.

In females, Zn levels pre-treatment ranged between 199.76 µg/dl and 484.07 µg/dl, while those post-treatment ranged between 296.75 and 521.35 µg/dl.

### 3.2 Malondialdehyde assay

**Fig. 8:**
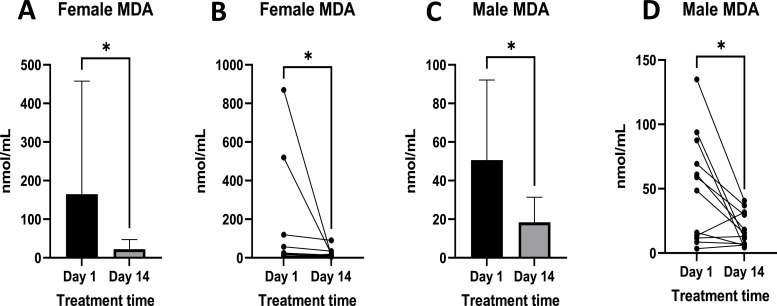
Shows MDA levels pre- and post-treatment in females (Fig. 8A and B) and males (Fig. 8C and D). Fig. 8A and C show mean ± standard deviation MDA blood plasma levels, while B and D show individual responses. Females n =10, Males *n =* 12. Where p-value * = <0.05.

Malondialdehyde (MDA) levels were statistically significantly reduced in both males and females (Fig. 8). Levels of MDA ranged from 6.27 nmol/ ml to 869 nmol/ ml to a range of 4.30 nmol/ml to 90.2 nmol/ ml (Fig. 8B) in females. There was a reduction in the standard deviation and mean levels post-treatment in both females and males (Fig. 8A and C). In males, levels ranged from 3.42 nmol/ ml to 134.88 nmol/ ml pre-treatment to 4.30 nmol/ml to 40.68 nmol/ ml post-treatment (Fig. 8D).

### 3.3δ-aminolaevulinic acid dehydratase activity

**Fig. 9:**
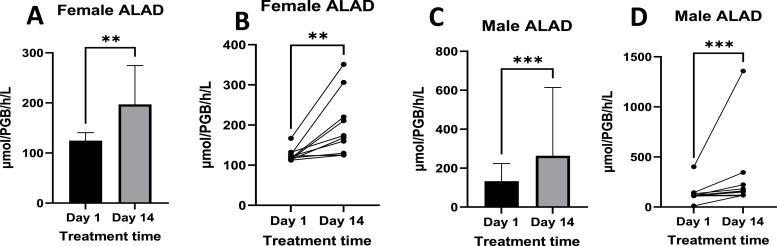
Shows the activity of δ-aminolaevulinic acid dehydratase pre- and post-treatment in females (Fig. 9A and B) and males (Fig. 9C and D). Fig. 9A and C show mean ± standard deviation ALAD blood plasma activity levels, while Fig. 8B and D show individual responses. Females n =10, Males *n =* 12. Where p-value * = <0.05.

The activity of δ-ALAD significantly increased by day 14 of treatment compared to baseline (day 1), as illustrated in Fig. 9. Statistical analysis revealed a marked difference in enzyme activity pre- and post-treatment in both sexes. In females, δ-ALAD activity prior to treatment ranged from 112.45 to 166.94 µmol PGB/h/L, increasing post-treatment to a range of 125.20 to 351.26 µmol PGB/h/L. In males, baseline activity varied widely from 11.36 to 402.28 µmol PGB/h/L, with post-treatment levels rising substantially to between 113.61 and 1357.54 µmol PGB/h/L.

### 3.4 Cortisol levels

**Fig. 10:**
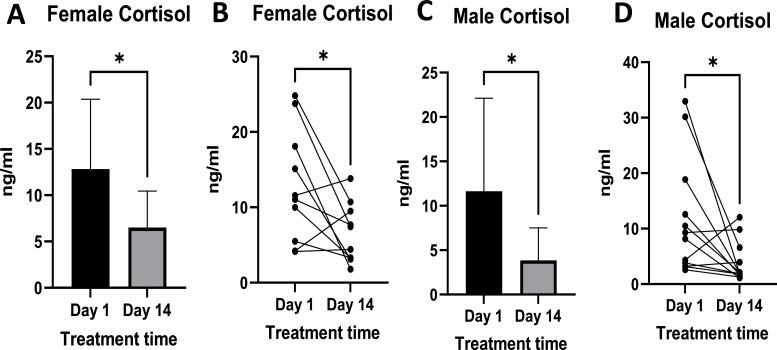
Shows the levels of cortisol pre- and post-treatment in females (Fig. 10A and Fig. 10B) and males (Fig. 10C and Fig. 9D). Fig. 10A and C show mean ± standard deviation, plasma cortisol levels, while Fig. 10B and D show individual responses. Females n =10, Males *n =* 12. Where p-value * = <0.05. Data is expressed as mean ± standard deviation.

Cortisol levels in the females ranged from 4.14 ng/ml to 24.82 ng/ml on the first day of the study and on day 14 ranged from 1.79 ng/ml to 13.82 ng/ml (Fig. 10B). There was a reduction in the mean levels and standard deviation of cortisol post-treatment; these changes were statistically significant (Fig. 10A).

In males, post-treatment levels ranged from 1.12 ng/ml to 12.03 ng/ml, and these were statistically significantly different from pre-treatment levels, which ranged from 3.21 ng/ml to 32.94 ng/ml (Fig. 10D). Like in females, the standard deviation and mean levels reduced post-treatment (Fig. 10C)

### 3.5 Blood urea nitrogen and creatinine

**Fig. 11:**
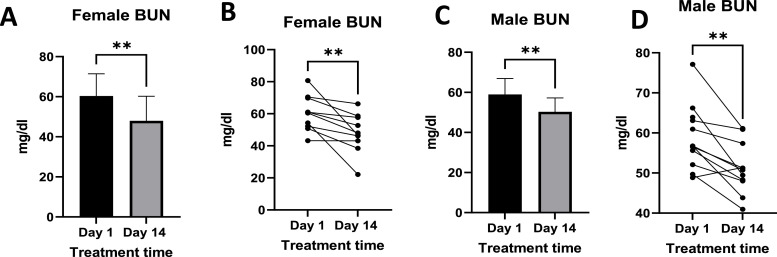
Shows the levels of blood urea nitrogen (BUN) pre- and post-treatment in females (Fig. 12A and Fig. 12B) and males (Fig. 12C and Fig. 12D). Fig. 12A and C show mean ± standard deviation, plasma BUN levels. In contrast, Fig. 12B and Fig. 12D show individual responses. Females n =10, Males *n =* 12. Where p-value ** = <0.05.

Plasma levels of BUN ranged from 43.24 mg/ dl to 80.70 mg/dl pre-treatment and 22.11 mg/dl to 66.17 mg/dl post-treatment (Fig. 11A and B) in females. In the males, levels pre-treatment ranged from 48.84 mg/dl to 61.13 mg/dl, while the post-treatment levels ranged from 39.92 mg/dl to 60.89 mg/dl (Fig. 11A and B). There was a significant reduction in the BUN when the two time points were compared in both males and females.

**Fig. 12:**
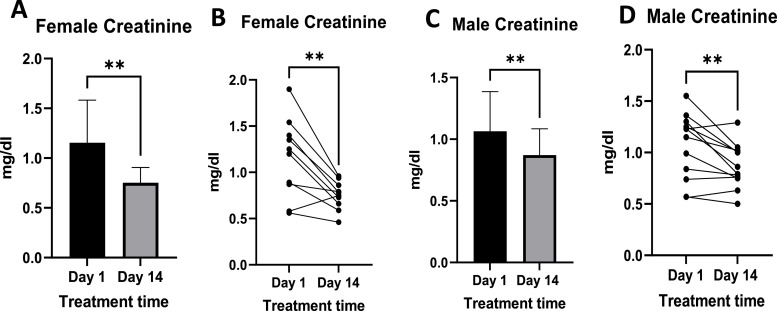
Displays the levels of creatinine pre- and post-treatment in females (Fig. 12A and Fig. 12B) and males (Fig. 12C and Fig. 12D). Fig. 12A and C illustrate mean plasma creatinine levels. In contrast, Fig. 12B and D show individual responses. Females n =10, Males *n =* 12. Where p-value ** = <0.05.

Female plasma creatinine levels ranged from 0.56 mg/dl to 1.9 mg/dl before treatment and from 0.46 mg/dl to 0.96 mg/dl after treatment (Fig. 12B). In the males, creatinine ranged from 0.56 mg/dl to 1.55 mg/dl pre-treatment and from 0.50 mg/dl to 1.29 mg/dl (Fig. 12D). These values showed a statistically significant reduction when Cre was compared pre- and post-treatment in both males and females (Fig. 12A and C).

## 4 Discussion

L-ascorbic acid administration in dogs in our study reduced MDA, BUN, CRE, and metal levels while increasing δ-ALAD activity in dogs living in a metal-polluted environment without withdrawal of environmental exposure.

### 4.1 Antioxidant properties of L-ascorbic acid

Lead exposure is known to elevate reactive oxygen species (ROS) in renal tissues, leading to mitochondrial dysfunction and oxidative stress. These effects impair glucose and phosphate reabsorption while promoting the retention of urea, CRE, and uric acid. In this study, treatment with L-AA resulted in a reduction of plasma CRE and BUN levels, suggesting an improvement in renal function. Previous research has demonstrated that L-AA mitigates oxidative stress and inflammation in the kidneys, which may enhance the excretion of accumulated metals, a trend also observed in this study. However, while CRE levels fell within the normal reference range (<1.4 mg/dL), BUN levels remained elevated post-treatment (>30 mg/dL), indicating that renal recovery may have been partial or that other contributing factors, such as continued metal exposure, hydration status, protein intake and protein metabolisation, may have influenced BUN retention (Salazar, 2014; Sharma et al., 2015). Furthermore, Kuraeiad & Kotepui, (2021) reported that BUN was more sensitive to BLL than CRE in Pb- induced renal impairment assessment. Persistently elevated levels of BUN may suggest the presence of Pb-induced nephropathy.

Malondialdehyde is a product of lipid peroxidation (Cordiano et al., 2023), and a portion of it is excreted in urine (Tsikas et al., 2023). There is a correlation between high levels of Pb and high levels of MDA (Fanaei et al. 2014; Popovic et al. 2015; Yimcharoen et al. 2019). In this study, the reduction of MDA is two-fold; firstly, by increased excretion due to improved kidney function as described above, and by a reduction in production due to the reduction of lipid peroxidation by L-AA.

High levels of cortisol and corticosterone are observed as a response to the oxidative stress that Pb and other metals initiate in metal-exposed individuals. High levels of cortisol predispose individuals to cardiovascular disease (Iob and Steptoe 2019), obesity (Hewagalamulage et al., 2016), and autoimmune diseases (Sharif et al. 2018; Slominski et al. 2020).The overall reduction in oxidative stress in the body, demonstrated by a reduction in MDA, also implies there is a reduction of oxidative stress on adrenal glands, resulting in the reductions observed in this study (Beglaryan et al., 2024). Henceforth, it will lessen the predisposition of individuals to various disease conditions.

### 4.2 Chelating properties of L-ascorbic acid

Post-treatment analysis revealed a reduction in Pb concentrations, consistent with previous findings that L-AA enhances Pb elimination from the body (Lihm et al., 2013). The reductions were mostly observed in younger dogs, who also presented with the highest BLL, aligning with results reported by Toyomaki et al., (2020) and Langlois et al., (2017). Similarly, studies in human populations have shown that children tend to exhibit higher BLLs than adults (Jain, 2016; Levin et al., 2021; Yabe et al., 2020).

The pronounced Pb reduction in younger dogs may be attributed to their lower bone lead storage capacity (Rădulescu & Lundgren, 2019), as well as their more efficient drug absorption, distribution, and elimination compared to older animals (Perrie et al., 2012). These factors likely contributed to their enhanced responsiveness to treatment. L-AA functions similarly to chelating agents (Pareja, 2016), which have demonstrated greater efficacy in cases of acute Pb exposure (<1 year) than in chronic exposure (>1 year), due to the mobilisation of Pb from bone stores over time (Al-Ghafari et al., 2019; Mumtaz et al., 2020). This explains the heightened effectiveness of L-AA in younger animals with shorter exposure durations.

Although yard surface conditions varied, no statistically significant difference was observed in blood lead levels (BLL) between dogs residing in paved yards and those on bare soil on the first day of treatment. This may be attributed to wind-driven dispersion of contaminated dust, which can settle even in paved areas, diminishing the protective effect of surface coverage. Interestingly, treatment response appeared more consistent among dogs from paved yards, as evidenced by a reduction in standard deviation, suggesting less variability in individual outcomes. Dobrescu et al., (2022) highlighted a key limitation in using BLL as a sole indicator of soil remediation efficacy, noting that BLL may reflect historical exposure due to Pb stored in the bone. There is a need for a more comprehensive assessment when evaluating the success of the remediation.

In this study, Zn levels were significantly reduced in male dogs following treatment. Previous studies suggest that L-AA may interfere with Zn binding to intestinal metallothionein, potentially influencing its absorption (Grosicki, 2004). However, this interaction does not appear to affect overall dietary Zn levels, which are considered normal within the range of 200–500 µg/dl (Papich, 2016). These reference values were maintained in both male and female dogs, pre- and post-treatment in this study.

Interestingly, the absence of Zn reduction in female dogs may reflect a physiological difference in absorption. Armah (2016) reported that women tend to absorb more Zn than men, a trait that could confer a protective advantage in maintaining Zn homeostasis. While this observation may help explain the sex-specific differences noted in our study, further investigation is warranted to confirm the underlying mechanisms contributing to this phenomenon.

Copper levels in male dogs decreased following L-AA administration, consistent with previous findings (Kaźmierczak-Barańska et al., 2020; Padayatty & Levine, 2016; Spoelstra-de Man et al., 2018). In contrast, female dogs typically exhibit higher circulating copper levels than males, primarily due to elevated ceruloplasmin (Cp) concentrations influenced by oestrogen (Bost et al., 2016; Wapnir, 1998). L-ascorbic acid has been shown to modulate oestrogen activity positively (Bostanci, 2012; Michos et al., 2006) which may explain the observed post-treatment elevation in copper levels among females. When compared to baseline values, copper concentrations remained within the normal physiological range both before and after treatment, that is, between 20 µg/dL and 80 µg/dL (Talcott, 2013).

Therefore, the use of L-AA to alleviate the toxic effects of non-essential metals should be conducted with the close monitoring of essential metals, such that they do not fall below permissible limits in males.

Delta-aminolaevulinic acid dehydratase (δ-ALAD) activity significantly improves in individuals exposed to lead (Pb) following administration of L-ascorbic acid (L-AA), through a dual mechanism.

Firstly, thiol groups within the δ-ALAD structure are depleted under oxidative stress conditions (Gul et al., 2018), leading to the formation of disulfide bonds that inactivate the enzyme. L-AA administration facilitates the reduction of these disulfide bonds, restoring thiol groups and reactivating the enzyme, thereby enhancing δ-ALAD activity.

Secondly, L-AA contributes to a reduction in Pb levels, which in turn diminishes oxidative stress. This reduction further supports the recovery of δ-ALAD activity.

Although being described as a weak chelating agent, L-AA will reduce oxidative stress biomarkers, unlike traditional chelation therapy drugs, which only reduce metal levels (Waidande et al., 2025).

Children have comparable levels of BLL to dogs (Chen et al. 2023;Chen et al., 2022). Therefore, as they also have less bone storage than adults, L-AA might potentially alleviate the effects of metal-induced oxidative stress and reduce BLL. There are strong associations between oxidative stress and its role in cognitive decline (Fan et al., 2024; Franzoni et al. 2021; Ramírez Ortega et al. 2021), which is an effect of Pb, is worse in Children. Studies by Alhusaini et al. (2022), Sepehri and Ganji (2016), and Nam et al. (2018) reported an improvement in brain function when L-AA was used in rats. Thus, L-AA may aid during the critical brain developmental stages in young children.

Finally, the therapeutic effects of L-AA observed in this study were primarily attributed to its antioxidant properties rather than to direct chelation or reduction of metals and metalloids. The decline in metal concentrations is likely a secondary outcome of reduced oxidative stress. The reduction in toxic metal levels was more pronounced for physiologically abundant elements such as lead (Pb), which lacks a defined safe threshold (WHO, 2024). Importantly, neurological impairments may persist even after Pb concentrations return to normal levels (Banda et al., 2024). In contrast, elements typically present at lower concentrations, such as arsenic (As) and cadmium (Cd), remained within permissible limits throughout the study, that is, mean values of about 0.1 µg/dL and <0.04 µg/dL, respectively (ATSDR, 2023) .

### 4.3 Study limitations

This study uses a within-subjects design, which means there was no control group. It has the disadvantage of reduced internal validity, as we are unable to fully isolate the effects of L-AA supplementation. Kidney function cannot be reliably assessed using blood plasma concentrations of blood urea nitrogen (BUN) and creatinine (CRE) alone; a comprehensive evaluation requires urinalysis to account for renal excretory performance and potential subclinical abnormalities. A major limitation of this study is the lack of longitudinal follow-up, which restricts our ability to evaluate the persistence and long-term impact of the observed effects. Furthermore, the absence of stratification by weight, age, and breed introduces potential confounding variables, as these factors may significantly influence physiological responses to environmental exposures and therapeutic interventions. Although all participants originated from similar household settings, variations in diet and environment, such as proximity to the former mining area, may contribute to differential outcomes and should be considered in future analyses. Due to inconsistencies in existing literature regarding baseline levels of MDA, δ-ALAD, and cortisol, direct comparisons with the data obtained in this study were not feasible.

## 5 Conclusion

The use of L-AA in dogs to mitigate the effects of lead (Pb) and other metals presents a promising treatment approach for animals residing in metal-contaminated areas. Its availability makes this method both cost-effective and accessible. The treatment appears to be more effective in younger individuals, with observed reductions in Pb levels. We hypothesise that L-AA supplementation may help alleviate the toxic effects of Pb and other metals by reducing susceptibility to oxidative stress-related conditions.

This finding suggests potential relevance for use in younger populations, as Pb levels in children are often similar to those observed in dogs. Given that children are particularly vulnerable to the adverse effects of Pb exposure, this approach may offer a pathway to improving their quality of life. However, careful monitoring of essential metals during treatment is crucial to avoid unintended imbalances. Further research is required to validate these findings, ideally using a between-subjects design with a larger sample size and comparative analysis with established chelating agents and long-term L-AA administration.

## Funding and acknowledgements

This work was supported by JST SPRING; Grant Number JPMJSP2119, the WISE Fellowship of the Department of Science and Technology, the Grant-in-Aid for Challenging Research (Pioneering); proposal number 20K20633 (SMMN), the Grant-in-Aid for Scientific Research(B); proposal number 23H03545, 23K28235 (SMMN) and Nos. 21H04919, 22KK0163 (MI). The authors acknowledge financial support from the JST/JICA SATREPS (Science and Technology Research Partnership for Sustainable Development; No. JPMJSA1501) and aXis (Accelerating Social Implementation for SDGs Achievement; No. JPMJAS2001) funded by JST. This study was also financed by the JST AJ-CORE Project (MI), JSPS CORE to CORE program (MI, SMMN), Hokkaido University SOUSEI-TOKUTEI Specific Research Projects (MI), JSPS Bilateral Open Partnership Joint Research Projects (JPJSBP120209902; SMMN), the Japan Prize Foundation (SMMN), and JICA Technical Cooperation Project (SMMN).

**Data set availability**

• The datasets from this current study are available from the corresponding author upon reasonable request.

**Artificial Intelligence**

• Artificial intelligence was not used in generating the subject matter of this write up.

## Ethical statement

This study received clearance from the Zambian Ministry of Fisheries and Livestock and was carried out strictly per its regulations. Ethical approval clearance was obtained from the University of Zambia Biomedical Research Ethics Committee (UNZABREC) under reference number 5310-2024.

## CRediT authorship contribution statement

**Nelly Banda:** Writing – original draft, Visualization, Software, Resources, Project administration, Methodology, Investigation, Formal analysis, Data curation, Conceptualization. **Mahongo Selwa:** Investigation. **Rio Doya:** Investigation. **Nyein Chan Soe:** Investigation. **Andrew Kataba:** Investigation. **John Yabe:** Investigation. **Golden Zyambo:** Investigation. **Kaampwe Muzandu:** Investigation. **Yared Beyene Yohannes:** Investigation. **Yoshinori Ikenaka:** Investigation. **Mayumi Ishizuka:** Writing – review & editing, Supervision, Project administration, Funding acquisition, Conceptualization. **Shouta MM Nakayama:** Writing – review & editing, Supervision, Project administration, Funding acquisition, Conceptualization.

## Declaration of competing interest

The authors declare that they have no known competing financial interests or personal relationships that could have appeared to influence the work reported in this paper.

## References

[bib0001] Aaseth J., Ajsuvakova O.P., Skalny A.V., Skalnaya M.G., Tinkov A.A. (2018).

[bib0002] Abam E., Okediran B.S., Odukoya O.O., Adamson I., Ademuyiwa O. (2008). Reversal of ionoregulatory disruptions in occupational lead exposure by vitamin C. Environmental Toxicology and Pharmacology.

[bib0003] Ahmad F., Haque S., Ravinayagam V., Ahmad A., Kamli M.R., Barreto G.E., Ashraf Ghulam Md (2020). Developmental lead (Pb)-induced deficits in redox and bioenergetic status of cerebellar synapses are ameliorated by ascorbate supplementation. Toxicology.

[bib0004] Al-Ghafari A., Elmorsy E., Fikry E., Alrowaili M., Carter W.G. (2019). The heavy metals lead and cadmium are cytotoxic to human bone osteoblasts via induction of redox stress. PLoS ONE.

[bib0005] Alhusaini A.M., Fadda L.M., Alsharafi H., Alshamary A.F., Hasan I.H. (2022). L-ascorbic acid and curcumin prevents brain damage induced via lead acetate in rats: possible mechanisms. Developmental Neuroscience.

[bib0006] Armah S.M. (2016). Fractional zinc absorption for men, women, and adolescents is overestimated in the current dietary reference intakes. The Journal of Nutrition.

[bib0007] Assi M.A., Hezmee M.N.M., Haron A.W., Sabri M.Y.M., Rajion M.A. (2016). The detrimental effects of lead on human and animal health. Veterinary World.

[bib0008] ATSDR (2023). Cadmium toxicity: clinical assessment - laboratory tests | environmental medicine | ATSDR. Agency for Toxic Substances and Disease Registry.

[bib0009] Banda N., Soe N.C., Yabe J., Doya R., Yohannes Y.B., Ikenaka Y., Ishizuka M., Nakayama S.M.M. (2024). Sex dependent intergenerational effects of lead in mouse model. Scientific Reports.

[bib0010] Bandodkar A., Justin William B., Kannan T.A., Arun Prasad A., Bharathidasan M., Jayaprakash R., George R.S. (2016). Intra-articular injection of ascorbic acid and dexamethasone for management of osteoarthritis in dogs. Annual Research and Review in Biology.

[bib0011] Beck A.C., Lash E.M., Hack J.B. (2020). Environmental toxic exposures using companion animals as an indicator of Human toxicity: A case report and discussion. Journal of Emergency Medicine.

[bib0012] Beglaryan N., Hakobyan G., Nazaretyan E. (2024). Vitamin C supplementation alleviates hypercortisolemia caused by chronic stress. Stress and Health.

[bib0013] Bost M., Houdart S., Oberli M., Kalonji E., Huneau J.F., Margaritis I. (2016). Dietary copper and human health: current evidence and unresolved issues. Journal of Trace Elements in Medicine and Biology.

[bib0014] Bostanci (2012). The effects of ascorbic acid on the estrogen/progesteron levels in the isolated rabbit uterine muscle. Journal of Clinical Gynecology and Obstetrics.

[bib0015] Bruno F., Miller A., Bruschetta G., Nava V., Rifici C., Zappalà S., Licata P. (2025). Levels of mineral elements in different organs of dogs from the ionian-etnean volcanic area. Animals.

[bib0016] Cao Y., Skaug M.A., Andersen O., Aaseth J. (2015).

[bib0017] Chen X., Cao S.Z., Wen D., Zhang Y., Wang B., Duan X. (2023). Domestic dogs as sentinels of children lead exposure: multi-pathway identification and source apportionment based on isotope technique. Chemosphere.

[bib0018] Chen X., Duan X., Cao S.Z., Wen D., Zhang Y., Wang B., Jia C. (2022). Source apportionment based on lead isotope ratios: could domestic dog’s blood lead be used to identify the level and sources of lead pollution in children?. Chemosphere.

[bib0019] Cordiano R., Di Gioacchino M., Mangifesta R., Panzera C., Gangemi S., Minciullo P.L. (2023).

[bib0020] Dobrakowski M., Pawlas N., Kasperczyk A., Kozłowska A., Olewińska E., Machoń-Grecka A., Kasperczyk S. (2017). Oxidative DNA damage and oxidative stress in lead-exposed workers. Human and Experimental Toxicology.

[bib0021] Dobrescu A.I., Ebenberger A., Harlfinger J., Griebler U., Klerings I., Nußbaumer-Streit B., Chapman A., Affengruber L., Gartlehner G. (2022). Effectiveness of interventions for the remediation of lead-contaminated soil to prevent or reduce lead exposure - A systematic review. Science of The Total Environment.

[bib0022] Doya R., Nakayama S.M.M., Nakata H., Toyomaki H., Yabe J., Muzandu K., Yohannes Y.B., Kataba A., Zyambo G., Ogawa T., Uchida Y., Ikenaka Y., Ishizuka M. (2020). Land use in habitats affects metal concentrations in wild lizards around a former lead mining site. Environmental Science and Technology.

[bib0023] El-Sebeay, A. S., Ibrahim, A. F., & Yousif, A. B. (2017). Effect of ascorbic acid on reproductive function of male rats exposed to lead acetate. In *Journal of High Institute of Public Health* (Vol. 47, Issue 2). www.jhiph.alexu.edu.eg.

[bib0024] Fan Z., Yang C., Qu X., Zhang J., Wu H., Yang Y., Huang Y., Zeng P., Xiang Z., Yang J. (2024). Association of oxidative stress on cognitive function: A bidirectional mendelian randomisation study. Molecular Neurobiology.

[bib0025] Fanaei, H., Khayat, S., Candidate, P. D., Halvaei, I., Ramezani, V., Azizi, Y., Kasaeian, A., Mardaneh, J., Parvizi, M. R., & Akrami, M. (2014). Effects of ascorbic acid on sperm motility, viability, acrosome reaction and DNA integrity in teratozoospermic samples. In *Iran J Reprod Med* (Vol. 12, Issue 2). PMC4009562 24799867

[bib0026] Franzoni F., Scarfò G., Guidotti S., Fusi J., Asomov M., Pruneti C. (2021).

[bib0027] Ghafouri-Fard S., Shoorei H., Mohaqiq M., Tahmasebi M., Seify M., Taheri M. (2021). Counteracting effects of heavy metals and antioxidants on male fertility. BioMetals.

[bib0028] Gordon D.S., Rudinsky A.J., Guillaumin J., Parker V.J., Creighton K.J. (2020). Vitamin C in health and disease: A companion animal focus. Topics in Companion Animal Medicine.

[bib0029] Gori E., Pierini A., Meucci V., Abramo F., Muscatello L.V., Marchetti V. (2021). Hepatic lead and copper concentrations in dogs with chronic hepatitis and their relationship with hematology, serum biochemistry, and histopathology. Journal of Veterinary Internal Medicine.

[bib0030] Grosicki A. (2004). Influence of vitamin C on cadmium absorption and distribution in rats. Journal of Trace Elements in Medicine and Biology.

[bib0031] Gul M., Bugday M.S., Erel O. (2018). Thiol–disulphide homoeostasis as an oxidative stress marker in men with varicocele. Andrologia.

[bib0032] Hegedus C., Andronie L., Uiuiu P., Jurco E., Lazar E.A., Popescu S. (2023).

[bib0033] Hewagalamulage S.D., Lee T.K., Clarke I.J., Henry B.A. (2016). Stress, cortisol, and obesity: a role for cortisol responsiveness in identifying individuals prone to obesity. Domestic Animal Endocrinology.

[bib0034] Iob E., Steptoe A. (2019). Cardiovascular disease and hair cortisol: a novel biomarker of chronic stress. Current Cardiology Reports.

[bib0035] Jain R.B. (2016). Trends and variability in blood lead concentrations among US children and adolescents. Environmental Science and Pollution Research.

[bib0036] Kaźmierczak-Barańska J., Boguszewska K., Adamus-Grabicka A., Karwowski B.T. (2020). Two faces of vitamin c—Antioxidative and pro-oxidative agent. Nutrients.

[bib0037] Kim J.H., Kang J.C. (2017). Effects of sub-chronic exposure to lead (Pb) and ascorbic acid in juvenile rockfish: antioxidant responses, MT gene expression, and neurotransmitters. Chemosphere.

[bib0038] Kim J.J., Kim Y.S., Kumar V. (2019). Heavy metal toxicity: an update of chelating therapeutic strategies. Journal of Trace Elements in Medicine and Biology.

[bib0039] Kuraeiad S., Kotepui M. (2021). Blood lead level and renal impairment among adults: A meta-analysis. International Journal of Environmental Research and Public Health.

[bib0040] Langlois D.K., Kaneene J.B., Yuzbasiyan-Gurkan V., Daniels B.L., Mejia-Abreuphd H., Frank N.A., Buchweitz J.P. (2017). Investigation of blood lead concentrations in dogs living in flint, Michigan. Journal of the American Veterinary Medical Association.

[bib0041] Lee J.W., Choi H., Hwang U.K., Kang J.C., Kang Y.J., Kim K.Il, Kim J.H (2019). Toxic effects of lead exposure on bioaccumulation, oxidative stress, neurotoxicity, and immune responses in fish: A review. Environmental Toxicology and Pharmacology.

[bib0042] Levin R., Zilli Vieira C.L., Rosenbaum M.H., Bischoff K., Mordarski D.C., Brown M.J (2021). The urban lead (Pb) burden in humans, animals and the natural environment. Environmental Research.

[bib0043] Li X., Hu F.X., Xu G. (2023). Membranous nephropathy caused by dimercaptosuccinic acid in a patient with Wilson’s disease: a case report and literature review. BMC Nephrology.

[bib0044] Liguori I., Russo G., Curcio F., Bulli G., Aran L., Della-Morte D., Gargiulo G., Testa G., Cacciatore F., Bonaduce D., Abete P. (2018).

[bib0045] Lihm H., Kim H., Chang H., Yoon M., Lee K., Choi J. (2013). Vitamin C modulates lead excretion in rats. Anatomy & Cell Biology.

[bib0046] Maruyama M., Ushine N., Watanabe Y., Ishii C., Saito K., Sakai H., Kuritani T., Doya R., Ogasawara K., Ikenaka Y., Yohannes Y.B., Ishizuka M., Nakayama S.M.M. (2024). Current situation of lead (Pb) exposure in raptors and waterfowl in Japan and difference in sensitivity to in vitro lead exposure among avian species. Environmental Pollution.

[bib0047] Michos C., Kiortsis D., Evangelou A., Karkabounas S. (2006). Antioxidant protection during the menstrual cycle: the effects of estradiol on ascorbic-dehydroascorbic acid plasma levels and total antioxidant plasma status in eumenorrhoic women during the menstrual cycle. Acta Obstetricia et Gynecologica Scandinavica.

[bib0048] Mumtaz S., Ali S., Khan R., Shakir H.A., Tahir H.M., Mumtaz S., Andleeb S. (2020).

[bib0049] Nam S.M., Chang B.J., Kim J.H., Nahm S.S., Lee J.H. (2018). Ascorbic acid ameliorates lead-induced apoptosis in the cerebellar cortex of developing rats. Brain Research.

[bib0050] Nam S.M., Cho I.-S., Seo J.S., Go T.-H., Kim J.-H., Nahm S.-S., Chang B.-J., Lee J.-H. (2019). Ascorbic acid attenuates lead-induced alterations in the synapses in the developing rat cerebellum. Biological Trace Element Research.

[bib0051] Padayatty S.J., Levine M. (2016). Vitamin C: the known and the unknown and goldilocks. Oral Diseases.

[bib0052] Papich M.G. (2016). Zinc. Saunders Handbook of Veterinary Drugs.

[bib0053] Pareja, M. (2016). *Relative antioxidant efficacy of α-tocopherol and ascorbic acid on blood lead, hemoglobin and hematocrit level of lead-exposed Rattus norvegicus (albino rat)*. https://www.researchgate.net/publication/296707152.

[bib0054] Pastorinho M.R., Sousa A.C.A., Pastorinho M.R., Sousa A.C.A. (2020). Pets as Sentinels, Forecasters and Promoters of Human Health.

[bib0055] Perrie Y., Badhan R.K.S., Kirby D.J., Lowry D., Mohammed A.R., Ouyang D. (2012). The impact of ageing on the barriers to drug delivery. Journal of Controlled Release.

[bib0056] Polymeropoulos E., Bagos P., Papadimitriou M., Rizos I., Patsouris E., Toumpoulis I. (2016). Vitamin C for the prevention of postoperative atrial fibrillation after cardiac surgery: A meta-analysis. Advanced Pharmaceutical Bulletin.

[bib0057] Popovic L.M., Mitic N.R., Miric D., Bisevac B., Miric M., Popovic B. (2015). Influence of vitamin c supplementation on oxidative stress and neutrophil inflammatory response in acute and regular exercise. Oxidative Medicine and Cellular Longevity.

[bib0058] Rădulescu A., Lundgren S. (2019). A pharmacokinetic model of lead absorption and calcium competitive dynamics. Scientific Reports.

[bib0059] Raeeszadeh M., Karimfar B., Amiri A.A., Akbari A. (2021). Protective effect of nano-vitamin C on infertility due to oxidative stress induced by lead and arsenic in male rats. Journal of Chemistry.

[bib0060] Ramírez Ortega D., González Esquivel D.F., Blanco Ayala T., Pineda B., Gómez Manzo S., Marcial Quino J., Carrillo Mora P., Pérez de la Cruz V. (2021).

[bib0061] Rendón-Ramírez A.L., Maldonado-Vega M., Quintanar-Escorza M.A., Hernández G., Arévalo-Rivas B.I., Zentella-Dehesa A., Calderón-Salinas J.V. (2014). Effect of vitamin E and C supplementation on oxidative damage and total antioxidant capacity in lead-exposed workers. Environmental Toxicology and Pharmacology.

[bib0062] Rizk M.A., Abdalla A.A., El-Sayed S.A.E.-S (2017). Evaluation of ascorbic acid in combination of ivermectin in augmentation the recovery from juvenile generalized demodicosis in dogs: A randomized clinical trial. PSM Veterinary Research.

[bib0063] Salazar J.H. (2014). Overview of Urea and Creatinine. Laboratory Medicine.

[bib0064] Sechi S., Flore F., Chiavollelli F., Dimauro C., Nudda A. (2017). Oxidative stress and food supplementation with antioxidants in therapy dogs. Canadian Veterinary Asscociation.

[bib0065] Sepehri H., Ganji F. (2016). The protective role of ascorbic acid on hippocampal CA1 pyramidal neurons in a rat model of maternal lead exposure. Journal of Chemical Neuroanatomy.

[bib0066] Sharif K., Watad A., Coplan L., Lichtbroun B., Krosser A., Lichtbroun M., Bragazzi N.L., Amital H., Afek A., Shoenfeld Y. (2018). The role of stress in the mosaic of autoimmunity: an overlooked association. Autoimmunity Reviews.

[bib0067] Sharma, A., Ahuja, A., Srivastava, M., & Kachhawa, J. P. (2015). Haemato-biochemical changes in dogs suffering from chronic renal failure. In *Indian Journal of Canine Practice* (Vol. 7, Issue 2).

[bib0068] Sies H. (2020). Oxidative stress: concept and some practical aspects. Antioxidants.

[bib0069] Sies, H., Berndt, C., & Jones, D. P. (2025). *Oxidative stress*. 17. 10.1146/annurev-biochem. 28441057

[bib0070] Slominski R.M., Tuckey R.C., Manna P.R., Jetten A.M., Postlethwaite A., Raman C., Slominski A.T. (2020). Extra-adrenal glucocorticoid biosynthesis: implications for autoimmune and inflammatory disorders. Genes & Immunity.

[bib0071] Smith D., Strupp B.J. (2013). The scientific basis for chelation: animal studies and lead chelation. Journal of Medical Toxicology.

[bib0072] Soe N.C., Yohannes Y.B., Kataba A., Tembo M., Yabe J., Zyambo G., Chawinga K., Muzandu K., Ikenaka Y., Ishizuka M., Nakayama S.M.M. (2024). Metals and arsenic distribution in stray dogs’ tissues around a lead–zinc mine in Kabwe, Zambia. Environmental Science and Pollution Research.

[bib0073] Spoelstra-de Man A.M.E., Elbers P.W.G., Oudemans-Van Straaten H.M. (2018). Vitamin C: should we supplement?. Current Opinion in Critical Care.

[bib0074] Talcott P.A. (2013). Copper. Small Animal Toxicology, Third Edition.

[bib0075] Tangahu B.V., Sheikh Abdullah S.R., Basri H., Idris M., Anuar N., Mukhlisin M. (2011). A review on heavy metals (As, Pb, and Hg) uptake by plants through phytoremediation. International Journal of Chemical Engineering.

[bib0076] Toyomaki H., Yabe J., Nakayama S.M.M., Yohannes Y.B., Muzandu K., Liazambi A., Ikenaka Y., Kuritani T., Nakagawa M., Ishizuka M. (2020). Factors associated with lead (Pb) exposure on dogs around a Pb mining area, Kabwe, Zambia. Chemosphere.

[bib0077] Tsikas D., Tsikas S.A., Mikuteit M., Ückert S. (2023).

[bib0078] van Eijkeren J.C.H., Olie J.D.N., Bradberry S.M., Vale J.A., de Vries I., Clewell H.J., Meulenbelt J., Hunault C.C. (2017). Modeling the effect of succimer (DMSA; dimercaptosuccinic acid) chelation therapy in patients poisoned by lead. Clinical Toxicology.

[bib0079] Waidande S.S., Kshirsagar M., Thorat V.M., Tiwari D.D. (2025). Role of antioxidant supplementation in enhancing chelation therapy for lead-induced oxidative stress in rats. Cureus.

[bib0080] Wang W., Hernandez J., Moore C., Jackson J., Narfström K. (2016). Antioxidant supplementation increases retinal responses and decreases refractive error changes in dogs. Journal of Nutritional Science.

[bib0081] Wani A.L., Ara A., Usmani J.A. (2015).

[bib0082] Wapnir R.A. (1998). Copper absorption and bioavailability. The American Journal of Clinical Nutrition.

[bib0083] WHO. (2024, September 27). *Lead poisoning*. https://www.who.int/news-room/fact-sheets/detail/lead-poisoning-and-health.

[bib0084] World Bank Group. (2020, December 17). *Zambia Mining and Environmental Remediation and Improvement Project*. https://www.worldbank.org/en/news/factsheet/2020/12/17/zambia-mining-and-environmental-remediation-and-improvement-project.

[bib0085] Yabe J., Nakayama S.M., Nakata H., Toyomaki H., Yohannes Y.B., Muzandu K., Kataba A., Zyambo G., Hiwatari M., Narita D., Yamada D., Hangoma P., Munyinda N.S., Mufune T., Ikenaka Y., Choongo K., Ishizuka M. (2020). Current trends of blood lead levels, distribution patterns and exposure variations among household members in Kabwe, Zambia. Chemosphere.

[bib0086] Yimcharoen M., Kittikunnathum S., Suknikorn C., Nak-On W., Yeethong P., Anthony T.G., Bunpo P. (2019). Effects of ascorbic acid supplementation on oxidative stress markers in healthy women following a single bout of exercise. Journal of the International Society of Sports Nutrition.

